# Use of Dietary Supplements and Influencing Factors in Children

**DOI:** 10.3390/ijerph21060734

**Published:** 2024-06-05

**Authors:** Orkut Koç, Merve Tosyalı, Şule Gökçe, Feyza Koç

**Affiliations:** Department of Pediatrics, Faculty of Medicine, Ege University, Children’s Hospital, 35100 Izmir, Turkey; orktkoc.f@gmail.com (O.K.); sule.gokce@ege.edu.tr (Ş.G.); feyza.koc@ege.edu.tr (F.K.)

**Keywords:** dietary supplement, family, vitamin–mineral, omega-3

## Abstract

Introduction: In recent years, the use of dietary supplements has increased in all age groups. Parents may also use these supplements for their children for different reasons. This study aims to determine the use of dietary supplements by children, the factors affecting this use, and the attitudes of parents about these products. Methods: A total of 1038 children aged 2–18 years without any chronic disease who presented to the pediatric outpatient clinics of Ege University Children’s Hospital were included in this study. Parents (*n* = 1000) who agreed to participate in the study were interviewed face-to-face, and a comprehensive questionnaire including questions about children’s use of dietary supplements, sociodemographic characteristics, and parents’ attitudes towards dietary supplements was administered. Analyses were performed with SPSS 25.0. Results: The mean age of the children included in our study was 8.6 ± 4.8 years, and 51% (*n* = 510) were male. It was found that 32.5% of the children used nutritional supplements, and vitamin–mineral preparations (23.2%) were the most frequently used. Omega-3 (19.3%) and immune support products (9.4%) were the second and third most frequently used supplements, respectively. A significant relationship was found between the use of dietary supplements and the child’s age, body weight, body mass index, parents’ educational level, being health worker, and economic status (*p* < 0.05). It was found that most of the families thought that vitamin–mineral and omega-3 products were beneficial for growth and development and that they received information from doctors most frequently before taking these products. However, it was found that families followed the media as the second most frequent source of information for these products. Conclusions: Approximately one-third of the children in our study use dietary supplements. It is very important to raise awareness among families about the use of these products when necessary and with the recommendation of a physician. To prevent families from using dietary supplements that are not necessary for their children, especially due to misinformation in the media, pediatricians should provide correct information to parents about these products at every clinic visit. A concerted effort is needed from policy makers, media organizations, and health care providers to guide the safe use of DS. The results obtained from this study will shed light on future randomized controlled prospective studies

## 1. Introduction

Dietary supplements (DS) are defined as products containing vitamins, minerals, plant extracts, amino acids, nutrients, or concentrates/combinations of any defined component intended to supplement the nutrients needed by the body and to support the diet [1,2]. The most common DS include vitamins–minerals, omega-3, immune support products (β glucan, Sambucus nigra, pelargonium sidoides, etc.), and probiotics [3].

Today, DS use in children and adults is widespread worldwide. According to estimates, almost 80% of the global population uses DS [4]. Although the United States of America has the highest consumption of DS, Italy, Russia, and Germany are the top countries in Europe in terms of DS consumption [5]. The use of dietary supplements in children has been reported to be 7.6–37% in studies conducted in different countries [1,6,7,8,9]. On average, one-third of children and adolescents in the United States take dietary supplements [10]. Other nations with high supplement use rates include China and Australia, where 22.6% and 32.4% of children use supplements, respectively [11]. DSs are widely used in our country as well as all over the world. One of the limited studies conducted in our country found that 23.9% of children aged 0–5 years and 3.1% of children aged 6–11 years used DSs [12]. Another study evaluated only children aged 15–18 years and found that 6.1% of them took these supplements [13]. In these studies, multivitamins and minerals are the most commonly used products [14].

Many factors can influence DS use, which may vary across countries. In studies from many different countries, factors that may affect DS use in the child age group include family income, parent’s education level, place of residence, parents’ occupational status, and family structure [14,15,16].

Routine use of DSs is not recommended in healthy children older than 1 year of age and adolescents who have a variety of nutrients in their diets [3,17]. However, families may be taking dietary supplements to strengthen immunity and support growth and development in their children without a physician’s recommendation [18,19,20]. It is known that during the COVID-19 pandemic period, the uncontrolled use of DS products in children and adults without a doctor’s recommendation increased considerably [21]. The main reasons for their use are to promote healthy growth and development by supporting diet, to reduce the risk of health problems, or to strengthen immunity [20]. Excessive intake of DSs can have several adverse health effects on the body, and deficiencies of certain DSs, such as vitamins and minerals, can lead to many diseases [22,23]. Especially in underdeveloped and developing countries, certain age groups (children, adolescents, and the elderly) are at risk of certain nutritional deficiencies. Our country is one of the developing countries, and vitamin–mineral deficiencies such as vitamin D, iron, and iodine are very common in children. It is obvious how important these DSs, which need to be replaced in addition to nutrition, are for the healthy growth and development of children. For this reason, the Ministry of Health is actively implementing national iron and vitamin D supplementation programs for infants and children [24,25]. Despite active replacement programs, the fight against diseases such as rickets, iron deficiency anemia, and endemic goiter, which occur due to vitamin and mineral deficiencies, occupies an important place in our health system [26,27]. It is very important for families to use these supplements with a doctor’s suggestion since children may need different doses and contents compared to adults [6]. In addition, some dietary supplements, especially immune supplements, do not have an appropriate dose recommendation for children and should be used with caution.

This study was conducted in the childhood age group in Turkey. It aims to analyze in detail the rates of DS use, the factors affecting this use, and the attitudes of parents towards the use of these products.

## 2. Methods

Children aged 2–18 years (*n* = 1038) admitted to pediatrics outpatient clinics of Ege University Medical Faculty Children’s Hospital were included in the study. Children with a history of early-moderate premature birth (<36 weeks), intrauterine growth retardation, chronic disease, and long-term hospitalization were excluded from the study. The study was conducted according to the guidelines of the Declaration of Helsinki. The approval of the study was obtained from the Ege University Medical Research Ethics Committee (23-5T/31). The informed consent was obtained from all subjects involved in the study.

The parents (mother/father) of eligible children who agreed to participate in the study were interviewed face-to-face, and a questionnaire was administered (*n* = 1000). General characteristics of the children such as age, gender, birth weight, birth size, educational status, and sociodemographic characteristics such as family structure, place of residence, economic status, age, educational level, parents being health workers, and parents’ attitudes towards the use of DSs were recorded. The anthropometric measurements of the children participating in the study were performed by the same person with the appropriate technique and the same measuring instruments, and the current body weight and height values were recorded and body mass indexes were calculated. We defined the educational status of the parents in two groups as those who had completed primary and secondary school education between 1–8 years and those who had completed high school or higher education for 9 years or more. While evaluating the economic status, parents were asked, “How do you perceive your economic status?” The answers were recorded as normal, rich, and poor.

Children’s use of dietary supplements was divided into 3 groups according to parents’ responses: “Yes, I use it.” “No, I have never used it.” and “I have used it before.”. To be included in the “I have used it before” category, we set the criterion of having used these products for at least 1 month. The generic names of the products we obtained from parents who used DSs for their children were separated according to the content information, and DS users were evaluated in four groups as multivitamin–mineral, omega-3, immune support products (β glucan, Sambucus nigra, pelargonium sidoides, propolis, quercetin, resveratrol, etc.) and other support products (probiotic, pollen, carob molasses, echinacea, ginger, black cumin oil etc.).

It was also recorded whether the parents used DSs themselves. These families were asked questions about their attitudes and knowledge about how long and for what purpose they used DSs for their children, from whom they received information about the products before using them, and whether they benefited from their use.

According to the use of DSs, the general characteristics of the children included in the study such as age, gender, weight, height, educational status, and sociodemographic characteristics of the families were statistically evaluated.

Data were analyzed using SPSS (Statistical Package for the Social Sciences) 25.0 package program. Chi-square and Student t-tests were used for the relationship between variables. According to Fisher’s normal distribution test, the data were found to fit the normal distribution. Therefore, parametric tests were preferred. For all analyses, *p* < 0.05 was considered significant.

## 3. Results

The general characteristics of the children included in the study are shown in Table 1. The mean age of the children was 8.6 ± 4.8 years, and 510 (51%) were male and 490 (49%) were female. The body weight of the children was 32.4 ± 18 kg, height was 128.8 ± 27.2 cm, and body mass index was 17.8 ± 3.3 kg/m^2^.

The sociodemographic characteristics of the families are shown in Table 2. The age of the mothers was 36.4 ± 6.6 years, and 10.9% (*n* = 109) of them were health workers. The age of the fathers was 39.8 ± 7 years, and 5.2% (*n* = 52) of them were healthcare workers. In evaluating the educational status of the parents, it was found that 63.7% of the mothers and 66.1% of the fathers had high school education or higher. A total of 868 families (86.6%) reported that they perceived their economic situation to be “normal”. In addition, it was determined that 7.5% (*n* = 75) of the parents used dietary supplements for themselves (Table 2).

**Figure 1:**
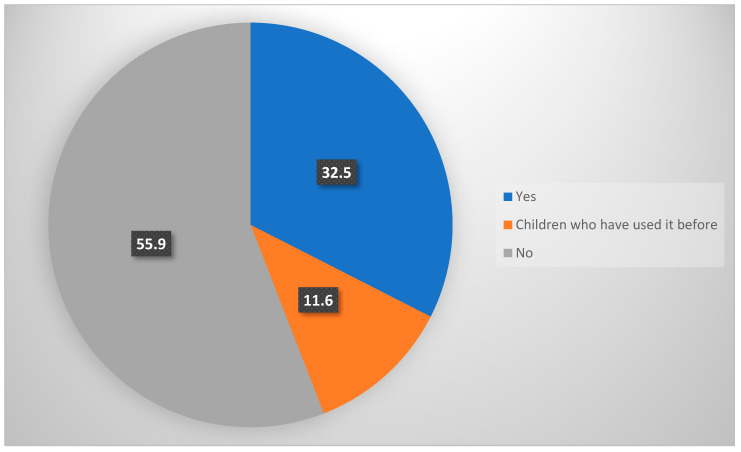
Dietary supplements use of the children included in the study (%).

When the use of dietary supplements was evaluated, it was found that 32.5% of the children were currently using these supplements, 55.9% had never used them, and 11.6% had used them before (Figure 1). The use of a supportive product was assessed according to the general characteristics of the children (Table 3). A statistically significant difference was found according to age, weight, height, body mass index, and siblings. It was determined that children with younger mean age, lower weight, height, and lower body mass index, and those who were the only child in the family used dietary supplements significantly more (*p* = 0.001, *p* = 0.009, *p* = 0.028, *p* = 0.001, *p* < 0.001, respectively).

**Figure 2:**
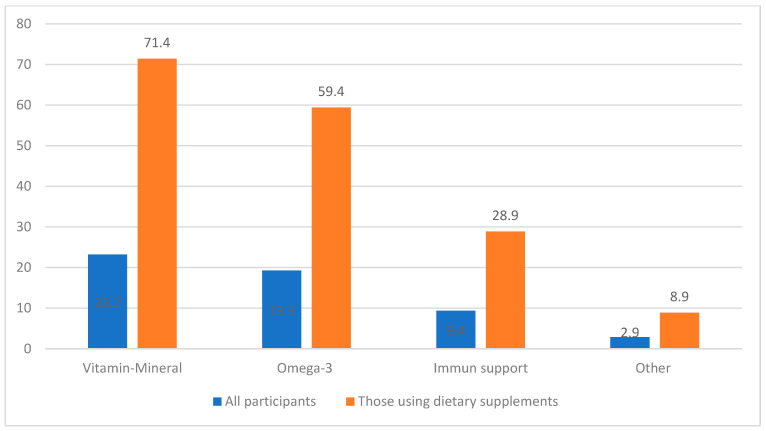
Distribution of dietary supplements (%) (distribution among all participants and those using dietary supplements, participants are using more than one product group).

Sociodemographic characteristics of the families according to children’s use of dietary supplements are evaluated in Table 4. A statistically significant difference was found between children’s use of supportive products and family structure, education of parents, whether they are a health worker, economic status, and their consumption of supportive products. It was determined that children with nuclear-type family structure (34.6%, *n* = 287) consumed more supportive products (*p* < 0.001). The rate of supplementary product use was found to be lower in children whose parents’ educational level was secondary school and below (*p* < 0.001). While 44% (*n* = 48) of the children whose mothers were health workers used these products, 41.3% (*n* = 45) did not use any (*p* = 0.005). On the other hand, 48.1% (*n* = 25) of the fathers who were health workers consumed DSs while 36.5% (*n* = 19) did not (*p* = 0.015). Children from families with normal and high economic status took supplements comparatively more than children from poor families (33.6%, 73.7%, 16.8%, respectively) (*p* < 0.001). A total of 77.3% (*n* = 58) of the parents who used DSs for themselves also used them for their children. The rate of parents taking DSs for themselves and giving them to their children was higher than those who did not use any (*p* < 0.001). Figure 2 shows which DSs are used by children. A total of 49.9% of them were using more than one DS at the same time. Among all the children included in the study, 23.2% (*n* = 232) were using vitamins and minerals, 19.3% (*n* = 193) used omega-3, 9.4% (*n* = 94) used immune support products, and 2.9% (*n* = 29) were using other supplements. For the children taking dietary supplements, these rates were 71.4%, 59.4%, 28.9%, and 8.9%, respectively (Figure 2). Regarding the children who used vitamin–mineral products, 53.9% took multivitamin–mineral, 17.2% vitamin D, and 13.4% multivitamin–mineral–omega 3 combinations. In the group of children using immune support products (*n* = 94), 32% used β glucan and 26.6% used propolis. When questioned about the use of other dietary supplements, it was found that carob molasses was 37.9%, probiotic was 20.9%, pollen was 20.9%, and royal jelly was 13.7%. When the mean ages of the children were analyzed according to the DS groups, the mean ages were found to be 7.5 ± 4.8 years, 8.5 ± 4.7 years, and 6.5 ± 3.3 years in the groups using vitamin–mineral, omega-3, and immune-supportive products, respectively. The mean age of children who used omega-3 was found to be higher than the others (*p* < 0.05).

The knowledge and attitudes of the families regarding their children’s use of dietary supplements are shown in Table 5. These families who consumed vitamin–mineral and omega-3 products stated that they used these products mostly for growth and development and thought that they had a positive effect on growth and development, while those who used immune support and other products stated that the reason for using these products was to protect health and added that they visited the doctor less this year compared to last year due to illness and that they thought that these products were beneficial (strengthened immunity) (Table 5). In addition, 16.2% of the families with children using omega-3 also said that the product increased school success. Most of the families thought that a doctor should be consulted when using DSs (vitamin–mineral: 94.8%, omega-3: 94.3%, immune support: 94.7%, other: 55.2%). Similarly, most of the families said that they received information about using vitamin–mineral, omega-3, and immune support products mostly from doctors (vitamin–mineral: 61.2%, omega-3: 49.2%, immune support product: 69.1%). It was found that these families used the media as the second most frequent source of information. However, most of the families using other support products (55.2%) received information from people other than doctors (relatives/neighbors/friends). Among parents who used other DS for their children, 69% said they did not think overuse would be a problem.

## 4. Discussion

Although the routine use of DSs in healthy children is not recommended, there has been an increase in recent years, especially with the COVID-19 pandemic [14,21]. The results of the study show that in this group of healthy children aged 2–18 years in Turkey, one-third of the participants (32.5%) were actively taking DSs.

The 2017–2018 National Health and Nutrition Examination Survey (NHANES) estimated the prevalence of supplement use in the past 30 days to be 34.0% among 3683 children and adolescents in the USA [14]. In studies conducted in different countries in the literature, DS use in children was found to be 32–37%, similar to the result in our study [1,8,28]. In another comprehensive study, 34.1% of 1431 children were reported to use these products [29].

In a few studies conducted in our country that examined a limited age group, it was reported that children used DS at rates ranging from 6.1% to 23.9% [12,13]. However, these studies did not analyze different age groups, socio-demographic and economic factors that may affect DS use, and parental attitudes toward DS use.

A child’s young age, low body weight, and body mass index were noticed as the factors that increased the use of supplementary products in our study. Similar results were reported in other studies [7,8,12,13,29,30]. In support of this result, the families stated that they mostly used vitamins, minerals, and omega-3 to support growth development and that the products were beneficial. In our study, it was found that immune support products and other products were mostly used for the treatment of diseases and the protection of health. In other studies, it has been shown that the most common reason for use is to improve general health, to support diet, to strengthen immunity, and to support physical growth [6,8,18,31]. An article on the use of DSs by children aged 3 to 12 in Poland found that 54.8% of caregivers increased their children’s intake of vitamins and minerals to boost immunity against flu and infections and to protect against the negative effects of antibiotics [32]. These products may originally be used to support the diet, but what is a striking finding in our study is that families prefer DS products less for this purpose. Dietary supplements may help to overcome deficiencies related to the nutritional problems of children. However, they are not recommended to be used instead of a healthy nutrition program.

In our study, it is reported that the most commonly used dietary supplements are vitamin–mineral, omega-3, and immune support products, respectively. In previous studies, it was found that vitamin–mineral use was most prevalent in children [1,18,28,29]. In a study conducted in the USA, it was reported that vitamin–mineral was first, immune support products second, and then omega-3 third in usage [28]. In recent years, there has been an increase in the use of omega-3 and immune support products. Although DSs (especially omega-3 and immune support products) are expensive products that are generally not covered by insurance, families may be willing to use them. It is known that parents who give DSs to their children tend to trust such products more than those who do not [32]. In our study, it is stated that parents with higher education levels, who are health workers, who have better economic status, and who also take DSs for themselves use more of these supplements for their children. For parents with higher family income who use a product for themselves, the rate of product use was found to be higher in their children as well [1,11,33]. It has been confirmed in our study that parents transfer their own behavioral patterns to their children, so those who use DSs themselves also give them to their children. Both the fact that it is easier for families with better economic status to buy DS and the desire of those who have observed a benefit in themselves to see the same effect in their children may bring these results.

When we asked parents about the effects of DS use on their children, they reported that they felt that children who used immune support products had stronger immunity, that most of them had been sick much less this year than last year and that infections healed more quickly. Similarly, parents reported that they felt that the use of DSs, mainly vitamin–mineral and omega-3 products, had a positive impact on growth and development. This information may indicate parents’ motivation for using DS products and their positive viewpoints and attitudes about these products. However, these results do not indicate that DS use contributes to the overall health and well-being of the child because the number of illnesses, duration of illness, duration of DS use, and dose were not evaluated in the study.

It is very important for families to obtain information about DSs from the right person. In our study, most of the families thought that a doctor should be consulted. Although most of the families received information from doctors for vitamin–mineral, omega-3, and immune support products, the source of information for other support products was mostly friends/neighbors. It was found that the second most common source of information for all DS was the media. A total of 72% of the families thought that the overuse of other dietary supplements was not harmful. In previous studies, it was reported that 18% of the families received information from healthcare professionals and 30.8% received it from physicians or pharmacists [8,9]. The use of these products should be determined according to the child’s age, health status, nutritional habits, and physician recommendations [18]. In addition, it is important to obtain them from reliable sources and to use them in appropriate doses. There are no adequate efficacy studies and appropriate dosage recommendations for most of the supplements other than vitamins, minerals, and omega-3 for children.

The strengths of this study are that it evaluated the use of DS in children with a large number of participants from different age groups, examined the anthropometric and sociodemographic factors that may affect this use in detail, and analyzed the reasons, attitudes, and awareness levels of parents to give DS to their children in detail. The findings obtained enable targeted interventions by determining the factors affecting the use of DS products in children and the attitudes of parents.

In conclusion, approximately one-third of the children who participated in our study use DS. A significant relationship was found between the use of DS and the child’s age, body weight, height, and body mass index, as well as the parents’ economic and educational level, whether they are a health worker, and dietary supplement usage for themselves. The most preferred DSs are vitamin–mineral, omega-3, and immune support products, respectively. It is necessary to consult a doctor before using any supplementary product. To prevent families from using DSs that are not necessary for their children, especially due to misinformation in the media, pediatricians should provide correct information to parents about these products at every clinic visit. A concerted effort is needed from policy makers, media organizations, and health care providers to guide the safe use of DSs.

This study will be very valuable as it is the first comprehensive study to examine in detail the rates of DS use in the childhood age group in Turkey, the factors affecting this use, and the attitudes of parents towards the use of these products. In order to determine the health effects of DS products in children, large prospective studies examining the dose of DSs, duration of use, reasons for concurrent hospital admission, frequency of hospital admission, and duration of illness are needed.

## 5. Limitations

The study was conducted in a tertiary university hospital covering a specific region and is therefore not representative of the whole population. Limitations of this study include the heavy reliance on parents’ recollections of children’s DS use. Children’s dietary intake, daily doses of DS, and duration of use were not recorded, which is another limitation of the study.

## Figures and Tables

**Table 1:** The general characteristics of the children included in the study (*n* = 1000).

Age * (year)		8.6 ± 4.8
Gender **	Male	510 (51)
	Female	490 (49)
Week of birth *		38.3 ± 2
Birth weight * (gr)		3181.9 ± 536.3
Birth height * (cm)		49.5 ± 2.6
Weight * (kg)		32.4 ± 18
Height * (cm)		128.8 ± 27.2
Body mass index * (kg/m^2^)		17.8 ± 3.3
Sibling **	Yes	696 (69.6)
	No	304 (30.4)
Educational status **	Not going to school	273 (27.3)
	Going to school	727 (72.7)

* Mean ± SD, ** *n* (%).

**Table 2:** The sociodemographic characteristics of families, *n* (%).

Family structure	Nuclear	830 (83)
	Extended	127 (12.7)
	Broken	43 (4.3)
Place of residence	City	885 (88.5)
	Village/town	115 (11.5)
Age of mothers * (year)	36.4 ± 6.6	
Education level of mothers (year)	1–8	363 (36.3)
	≥9	637 (63.7)
Mother’s being a healthcare worker	Yes	109 (10.9)
	No	891 (89.1)
Age of fathers * (year)	39.8 ± 7	
Education level of fathers (year)	1–8	339 (33.9)
	≥9	661 (66.1)
Father’s being a healthcare worker	Yes	52 (5.2)
	No	948 (94.8)
Economic status	Normal	868 (86.8)
	Poor	113 (11.3)
	Rich	19 (1.9)
Parent’s use of supplements	Yes	75 (7.5)
	No	925 (92.5)

* Mean ± SD.

**Table 3:** Evaluation of the general characteristics of the children according to the use of dietary supplements.

		Use of Dietary Supplements			*p*
		No *n* = 559	Yes *n* = 325	Used it before *n* = 119	
Age * (year)		9 ± 4.8	7.8 ± 4.7	8.3 ± 4.6	0.001
Gender **	Male	280 (54.9)	175 (34.3)	55 (10.8)	0.4
	Female	279 (56.9)	150 (30.6)	61 (12.4)	
Week of birth *		38.4 ± 1.8	38.3 ± 2.1	38 ± 2.3	0.2
Birth of weight * (gr)		3189.8 ± 517.7	3186.1 ± 550.3	3129.4 ± 585.4	0.5
Birth of height * (cm)		49.5 ± 2.3	49.6 ± 2.8	49.1 ± 3	0.2
Weight * (kg)		33 ± 18.2	30.4 ± 18	30.7 ± 16.3	0.009
Height * (cm)		130.8 ± 27	125 ± 27.7	127 ± 26.2	0.028
Body mass indeks * (kg/m^2^)		18.1 ± 3.51	17.3 ± 3	17.5 ± 2.8	0.001
Sibling **	Yes	430 (61.8)	194 (27.9)	72 (10.3)	<0.001
	No	129 (42.4)	131 (43.1)	44 (14.5)	
Educational status **	Going to school	405 (55.7)	236 (32.5)	86 (11.8)	0.9
	Not going to school	154 (56.4)	89 (32.6)	30 (11)	

* Mean ± SD, ** *n* (%).

**Table 4:** Evaluation of sociodemographic characteristics of the families according to children’s use of dietary supplements, *n* (%).

		Use of Dietary Supplements			*p*
		No *n* = 559	Yes *n* = 325	Used it before *n* = 119	
Family structure	Nuclear	440 (53)	287 (34.6)	103 (12.4)	<0.001
	Extended	96 (75.6)	24 (18.9)	7 (5.5)	
	Broken	23 (53.5)	14 (32.6)	6 (14)	
Place of residence	City	485 (54.8)	296 (33.4)	104 (11.8)	0.14
	Village/town	74 (64.3)	29 (25.2)	12 (10.4)	
Age of mothers (year) *		36.6 ± 6.7	35.9 ± 6.4	36.9 ± 6.4	0.2
The education level of mothers (year)	1–8	268 (73.8)	53 (14.6)	42 (11.6)	<0.001
	≥9	291 (45.7)	272 (42.7)	74 (11.6)	
Mother’s being a healthcare worker	No	514 (57.7)	277 (31.1)	100 (11.2)	0.005
	Yes	45 (41.3)	48 (44)	16 (14.7)	
Age of fathers (year) *		40.4 ± 7.2	38.8 ± 6.7	39.7 ± 7	0.005
The education level of fathers (year)	1–8	246 (72.6)	63 (18.6)	30 (8.8)	<0.001
	≥9	313 (47.4)	262 (39.6)	86 (13)	
Father’s being a healthcare worker	No	540 (57)	300 (31.6)	108 (11.4)	0.015
	Yes	19 (36.5)	25 (48.1)	8 (15.4)	
Economic status	Normal	471 (54.3)	292 (33.6)	105 (12.1)	<0.001
	Poor	83 (73.1)	19 (16.8)	11 (9.7)	
	Rich	5 (26.3)	14 (73.7)	0 (0)	
Parent’s use of supplements	No	552 (59.7)	267 (28.9)	106 (11.5)	<0.001
	Yes	7 (9.3)	58 (77.3)	10 (13.3)	

* Mean ± SD.

**Table 5:** The knowledge and attitudes of the families regarding their children’s use of dietary supplements, (*n*, %).

		Vitamin-Mineral	Omega-3	Immune Support	Other
What purpose do you use dietary supplements for your children?					
Growth and development		163 (70.3)	141 (73.1)	11(11.7)	18 (62.1)
The treatment of diseases		21 (9.1)	6 (3.1)	45 (47.9)	7 (24.1)
Protect health		157 (67.7)	123 (63.7)	88 (93.6)	19 (65.5)
Support nutrition		49 (21.1)	66 (34.2)	5 (5.3)	8 (27.6)
Who did you ask about dietary supplements before using them for your child?					
Pediatrician		142 (61.2)	95 (49.2)	65 (69.1)	11 (37.9)
Family doctor		83 (35.8)	77 (39.9)	23 (24.5)	3 (10.3)
Internet/TV/newspaper/magazine/social media		66 (28.4)	72 (37.3)	27 (28.7)	8 (27.6)
Relatives/neighbors/friends		30 (12.9)	38 (19.7)	17 (18.1)	16 (55.2)
Other		4 (1.7)	7 (3.6)	2 (2.1)	1 (3.4)
Do I need to consult a doctor?					
Yes		220 (94.8)	182 (94.3)	89 (94.7)	16 (55.2)
No		12 (5.2)	9 (5.7)	5 (5.3)	13 (44.8)
Is a blood test required before using these products?					
Yes		185 (79.7)	124 (64.2)	43 (45.7)	8 (27.6)
No		47 (20.3)	69 (35.8)	51 (54.3)	21 (72.4)
Can excessive use cause problems?					
Yes		199 (85.8)	117 (60.6)	54 (57.4)	9 (31)
No		33 (14.2)	76 (39.4)	40 (42.6)	20 (69)
Have these products been beneficial for your child?					
Yes		198 (85.3)	167 (86.5)	83 (88.3)	26 (89.7)
	Supported growth and development	110 (55.5)	98 (58.7)	2 (2.4)	8 (30.8)
	Immunity strengthened	73 (36.9)	42 (25.1)	81 (97.6)	17 (65.4)
	Increased school success	15 (7.6)	27 (16.2)	-	1 (3.8)
No		34 (14.7)	26 (13.5)	11 (11.7)	3 (10.3)

## Data Availability

The raw data supporting the conclusions of this article will be made available by the authors on request.

## Abbreviation

Dietary supplement (DS)

## References

[1] Dwyer J., Nahin R.L., Rogers G.T., Barnes P.M., Jacques P.M., Sempos C.T., Bailey R. (2013). Prevalence and predictors of children’s dietary supplement use: The 2007 National Health Interview Survey. Am. J. Clin. Nutr..

[2] Ministry of Food, Agriculture and Livestock Turkish Food Codex Supplementary Foods Communiqué 2013, Issue No: 28737. https://www.resmigazete.gov.tr/eskiler/2013/08/20130816-16.htm.

[3] Kleinman R.E., Coletta F.A. (2016). Historical Overview of Transitional Feeding Recommendations and Vegetable Feeding Practices for Infants and Young Children. Nutr. Today.

[4] Low T.Y., Wong K.O., Yap A.L.L., De Haan L.H.J., Rietjens I.M.C.M. (2017). The Regulatory Framework Across International Jurisdictions for Risks Associated with Consumption of Botanical Food Supplements. Compr. Rev. Food Sci. Food Saf..

[5] Statista. https://www.statista.com/statistics/589452/value-dietary-supplements-markets-europe-by-country.

[6] Perlitz H., Mensink G.B.M., Lage Barbosa C., Richter A., Brettschneider A.K., Lehmann F., Patelakis E., Frank M., Heide K., Haftenberger M. (2019). Use of vitamin and mineral supplements among adolescents living in Germany-Results from EsKiMo II. Nutrients.

[7] Jeon J.H., Seo M.Y., Kim S.H., Park M.J. (2021). Dietary supplement use in Korean children and adolescents, KNHANES 2015–2017. Public Health Nutr..

[8] Jun S., Cowan A.E., Tooze J.A., Gahche J.J., Dwyer J.T., Eicher-Miller H.A., Bhadra A., Guenther P.M., Potischman N., Dodd K.W. (2018). Dietary Supplement Use among U.S. Children by Family Income, Food Security Level, and Nutrition Assistance Program Participation Status in 2011–2014. Nutrients.

[9] Kobayashi E., Sato Y., Nishijima C., Chiba T. (2019). Concomitant Use of Dietary Supplements and Medicines among Preschool and School-Aged Children in Japan. Nutrients.

[10] European Food Safety Authority. https://www.efsa.europa.eu/en/topics/topic/food-supplements.

[11] Chen S., Binns C.W., Maycock B., Liu Y., Zhang Y. (2014). Prevalence of dietary supplement use in healthy pre-school Chinese children in Australia and China. Nutrients.

[12] Turkey Nutrition and Health Survey 2019. https://hsgm.saglik.gov.tr/depo/birimler/saglikli-beslenme-hareketli-hayat-db/Yayinlar/kitaplar/TBSA_RAPOR_KITAP_20.08.pdf.

[13] Turkey Nutrition and Health Survey 2010. https://hsgm.saglik.gov.tr/depo/birimler/saglikli-beslenme-hareketli-hayat-db/Yayinlar/kitaplar/diger-kitaplar/TBSA-Beslenme-Yayini.pdf.

[14] Stierman B., Mishra S., Gahche J.J., Potischman N., Hales C.M. (2020). Dietary Supplement Use in Children and Adolescents Aged ≤ 19 Years—United States, 2017–2018. MMWR Morb. Mortal. Wkly. Rep..

[15] Piórecka B., Koczur K., Cichocki R., Jagielski P., Kawalec P. (2022). Socio-Economic Factors Influencing the Use of Dietary Supplements by Schoolchildren from Małopolska Voivodship (Southern Poland). Int. J. Environ. Res. Public Health.

[16] Barretto J.R., Gouveia M.A.D.C., Alves C. (2024). Use of dietary supplements by children and adolescents. J. Pediatr..

[17] Kleinman R. (2009). Feeding the Child. Pediatric Nutrition Handbook.

[18] Liu H., Zhang S., Zou H., Pan Y., Yang Q., Ouyang Y., Luo J., Lin Q. (2019). Dietary Supplement Use Among Chinese Primary School Students: A Cross-Sectional Study in Hunan Province. Int. J. Environ. Res. Public Health.

[19] Panjwani A.A., Cowan A.E., Jun S., Bailey R.L. (2021). Trends in Nutrient- and Non-Nutrient-Containing Dietary Supplement Use among US Children from 1999 to 2016. J. Pediatr..

[20] Bailey R.L., Gahche J.J., Thomas P.R., Dwyer J.T. (2013). Why US children use dietary supplements. Pediatr. Res..

[21] U.S. Food and Drug Administration, Center for Food Safety and Applied Nutrition Questions and Answers on Dietary Supplements. https://www.fda.gov/food/information-consumers-using-dietary-supplements/questions-and-answers-dietary-supplements.

[22] Woźniak D., Przysławski J., Banaszak M., Drzymała-Czyż S. (2022). Dietary Supplements among Children Ages 0–3 Years in Poland-Are They Necessary?. Foods.

[23] Hunt K., Ernst E. (2011). The evidence-base for complementary medicine in children: A critical overview of systematic reviews. Arch. Dis. Child.

[24] Yalçın S.S., Buzgan T., Köse M.R., Altunsu A.T., Sahinli S., Tunç B., Köksal E., Ozbaş S., Pekcan G., Yurdakök K. (2013). A community-based iron supplementation program, “Iron-Like Turkey”, and the following prevalence of anemia among infants aged 12–23 months. Turk. J. Pediatr..

[25] Vitamin D Deficiency Prevention and Control Programme. https://hsgm.saglik.gov.tr/depo/birimler/cocuk-ergen-sagligi-db/Programlar/D_vitamini_Rehberi.pdf.

[26] Aksu T., Ünal Ş. (2023). Iron deficiency anemia in infancy, childhood, and adolescence. Turk. Arch. Pediatr..

[27] Koc F., Halicioglu O., Sutcuoglu S., Asik Akman S., Aksit S. (2014). Vitamin D supplementation during the first two years of life in Izmir, Turkey. Minerva Pediatr..

[28] Qato D.M., Alexander G.C., Guadamuz J.S., Lindau S.T. (2018). Prevalence of Dietary Supplement Use in US Children and Adolescents, 2003–2014. JAMA Pediatr..

[29] Namazi N., Kelishadi R., Heshmat R., Motlagh M.E., Sanaei M., Shafiee G., Ziaodini H., Beshtar S., Taheri M., Aminaee T. (2019). Determinants of taking dietary supplements in Iranian children and adolescents: The CASPIAN-V study. J. Diabetes Metab. Disord..

[30] O’Brien S.K., Malacova E., Sherriff J.L., Black L.J. (2017). The Prevalence and Predictors of Dietary Supplement Use in the Australian Population. Nutrients.

[31] C. S. Mott Children’s Hospital (2022). Healthy Eating and Use of Dietary Supplements in Children.

[32] Piekara A., Krzywonos M., Kaczmarczyk M. (2020). What Do Polish Parents and Caregivers Think of Dietary Supplements for Children Aged 3–12?. Nutrients.

[33] Sato Y., Yamagishi A., Hashimoto Y., Virgona N., Hoshiyama Y., Umegaki K. (2009). Use of dietary supplements among preschool children in Japan. J. Nutr. Sci. Vitaminol..

